# Genome Sequence of *Citrobacter* sp. CtB7.12, Isolated from the Gut of the Desert Subterranean Termite *Heterotermes aureus*

**DOI:** 10.1128/genomeA.01290-15

**Published:** 2015-11-05

**Authors:** Héctor Fontes-Perez, Myrna Olvera-García, America Chávez-Martínez, Felipe A. Rodriguez-Almeida, Claudio A. Arzola-Alvarez, Alejandro Sanchez-Flores, Agustín Corral-Luna

**Affiliations:** a Facultad de Zootecnia y Ecología, Universidad Autónoma de Chihuahua, Chihuahua, Chihuahua, México; b Unidad de Secuenciación Masiva y Bioinformática, Instituto de Biotecnología, Universidad Nacional Autónoma de México, Cuernavaca, Morelos, México

## Abstract

The draft genome of *Citrobacter* sp. CtB7.12, isolated from termite gut, is presented here. This organism has been reported as a cellulolytic bacterium, which is biotechnologically important because it can be used as a gene donor for the ethanol and biofuel industries.

## GENOME ANNOUNCEMENT

*Citrobacter* spp. are commonly found in water, soil, and gastrointestinal tracts of animals and humans. Recently, *Citrobacter* spp. have been isolated from the gut of terrestrial land slug (1), termite (2), and silkworm (3). In all of these cases, a cellulolytic activity has been recognized. Biofuel production represents a novel clean energy source that is growing quickly. Lignocellulosic biomass has been recognized as a potential low-cost source of soluble sugars used for fermentation to produce ethanol (4). However, the biomasses have to be pretreated with severe chemical compounds, increasing the economic and environmental costs. An alternative solution to this problem could be the use of bacterial cellulases. Termites have been proposed as a candidate donor due to their feeding habits. This insect can digest as much as 99% of cellulose. To achieve this, they use a wide variety of microorganisms inhabiting their gut, since these can produce and secrete an enzymatic complex involved in cellulose breakdown (4, 5).

We isolated *Citrobacter* sp. cellulolytic strain CtB7.12 from the gut of the *Heterotermes aureus* termite found in the north of Mexico. The strain shows significant cellulolytic activity in a Congo red assay and has potential biotechnological applications. The genome sequence may contribute to finding new cellulases with high enzymatic activity. Total DNA from *Citrobacter* sp. CtB7.12 was extracted using the NucleoSpin genomic DNA purification kit (Macherey Nagel, Düren, Germany). Illumina sequencing libraries were prepared following the vendor’s protocol, and a total of 1,987,790 reads (estimated coverage ~55×) were generated using the Illumina GAIIx platform. The assembly was performed with ABySS version 1.3.5, using a *k*-mer size of 49. An assembly of 5,019,613 bp in 53 contigs with lengths greater or equal to 1,000 bp was obtained with *N*_50_ and *N*_90_ values of 222,754 bp and 78,341 bp, respectively. The average contig length was 94,709 bp, giving a considerable space to search for genes. The average GC content was 53.25%. High-quality draft genomes were obtained using REAPR version 3, SSPACE version 3, and GapFiller version 1.10 (6–8) for misassembly error correction, scaffolding, and scaffold gap filling, respectively. Gene prediction was performed using GeneMark, giving a total of 4,643 protein-coding genes by intersecting all three predictions (9). The genome sequences were annotated using an adaptation from the Trinotate pipeline: Transcriptome Functional Annotation and Analysis (http://trinotate.github.io). Based on the 16S rRNA gene, *Citrobacter amalonaticus* was 99% identical to *Citrobacter* sp. strain CtB7.12. According to the Kostas Lab-ANI calculator (http://enve-omics.ce.gatech.edu/ani), the ANI (average nucleotide identity) value of *Citrobacter* sp. strain CtB7.12 compared to *C. amalonaticus* is 91.21%, which suggests that strain CtB7.12 and *C. amalonaticus* belong to different species.

Through the functional annotation of *Citrobacter* sp. CtB7.12, we identified some genes related to cellulose degradation, such as endo-1, 4-d-glucanases, cellulases, and beta-glucosidases, which could be used for enzymatic hydrolysis in a microbial pretreatment to break off cellulosic biomass.

### Nucleotide sequence accession numbers.

This whole-genome shotgun project has been deposited at DDBJ/EMBL/GenBank under the accession number LIFT00000000. The version described in this paper is the first version, LIFT01000000.
